# Annotations

**Published:** 1894-10-06

**Authors:** 


### Annotations.

### The Illness of the Tzar.

The Tzar is said to be suffering from nephritis, which has supervened upon an attack of influenza. The disease, in Professor Leyden's judgment, is in an advanced stage. "There can be no longer any doubt," it is stated, "that a change for the worse has taken place in the Tzar's condition, which gives cause for great anxiety." He is not only ill in body, but very depressed in mind. His Majesty, it is further stated, suffers great pain, looks very ill, and during the last few weeks has lost enormously in weight. These facts are of the very gravest import. Undoubtedly there are many cases of Bright's disease, even in its more chronic forms, which are compatible with life, and moderately comfortable life for many years; and in the Tzar's case, we have it on Professor Leyden's authority that there is no cause for immediate alarm. But there are two or three facts in connection with the illustrious patient's condition which have a very serious significance for the medical mind. Chief among these is the alarming loss of flesh; this, coupled with the appearance of distress and suffering, and the deep depression of spirits, gives a significance to the words "no immediate cause for alarm" which only the medical mind can fully appreciate. Undoubtedly, if the facts as reported in the newspapers be entirely correct, the gravest consequences can only be averted with infinite pains and difficulty, if at all. There is always, of course, the possibility that the facts, as reported in the newspapers, may not be entirely correct.

### The Teaching of Hygiene in Schools.

Not schoolmasters and school children only, but all sensible people, who read Sir Douglas Galton's address to the members of the Sanitary Institute on the "Teaching of Hygiene in Schools," will put down their newspapers with a sigh of relief. We are not, after all, to be asked to impose upon the poor little worried inmates of elementary schools a catechism or "whole duty of childhood" on the subject of abstract hygiene science. Sir Douglas Galton promised a paper on the question, and many persons could think of nothing else but the new burden which seemed to be about to add the last straw to the crushing load of elementary school life. The danger, happily, is past, and the children will now breathe again. But school hygiene is to be taught, notwithstanding; and as it is now proposed to be taught it will prove both interesting and of great practical and educational use. What is school hygiene? It consists in keeping the school premises and the children healthy, and that, in the last resort, means keeping them thoroughly sweet and clean. Sir Douglas Galton proposes to teach the children the science of "applied cleanliness," a phrase as excellent as the notion is sound. They are to be taught how to keep every part of the school clean, and that by the very practical method of cleaning it themselves. They are, moreover, to be drilled in the equally necessary art of keeping themselves clean, "themselves," including their persons and their clothing. It may seem as if Sir Douglas Galton were making a very great fuss about a very little when he read a grave paper to a grave scientific audience on so very obvious and elementary a matter. But in reality it was not so. For, as a matter of fact, elementary schools are not kept clean and sweet, nor are the children by any means as fresh and spotless in their persons and attire as they ought to be. And dirt, even in these forms, is poison, disease and death. By all means then let Sir Douglas Galton put forward his excellent ideas on the "teaching" of hygiene in schools. Their practicality will make them a relief from abstract and book study, and their utility both to the children, their teachers, and the ratepayers is quite undeniable.

### Ladies at Golf.

Last week the Annual Golf Ball at St. Andrews was opened by no less famous a person than Mr. A. J. Balfour, M.P. "Ladies at Golf" remind one of the famous picture of "Hunters at Grass," they themselves would make such a charming picture. For the ladies who play golf are almost always charming. They have the most natural and delightful of all charms, the charm of excellent health and the liveliest of spirits. Costume is always a matter of supreme importance to the more fascinating sex; and the light and easy but well-fitting costume which is appropriate for golf is exactly the costume which sets off to greatest advantage the square-shouldered, erect, muscular, and handsome young woman who finds her daily pleasure on the golf links. The practical physiologist, that is the reflecting person who believes that physiology was made for man and not man for physiology, takes large pleasure in the sight of perfectly formed and perfectly healthy humanity. Not the least of the fascinations of St. Andrews is a daily or bi-daily visit to the ladies' golf course. It will surprise many of the fair women of our more southern regions to be told that the lady golfers at the metropolis of golf are numbered by hundreds; and that, like the golfing men, they would be exceedingly difficult to match in the physiological perfection of their form, their lithe activity, and their natural and healthy beauty. Of all the ways of spending life the outdoor way is incomparably the best from the standpoint of physical perfection. The old-fashioned fortune-teller who used to teach her devotees how to become beautiful told them to wash their cheeks in the dew of the early May morning. The cunning witch who studies physiology in our own times will certainly tell them to play golf. It has often been asked how pleasant and youth-preserving employment might be found for the innumerable young women of the prosperous classes who are under no necessity to "toil or spin." The problem has been excellently solved at St. Andrews. Time never hangs heavily on the hands of the Clara Vere de Veres of that delightful home of golf and golfers. It is a sight never to be forgotten to look day by day on the fair forms and bright eyes of the frequenters of the ladies' course. Young men of the prosperous classes who are in search of healthy and beautiful wives with sweet tempers and wholesome minds cannot do better than wend their way to St. Andrews.

